# Research hotspots and trends on acupuncture treatment for headache: a bibliometric analysis from 2003 to 2023

**DOI:** 10.3389/fnins.2024.1338323

**Published:** 2024-03-21

**Authors:** Shun Zhao, Songfeng Hu, Yujing Luo, Wangjun Li, Fenfen Zhao, Changkang Wang, Fanlei Meng, Xingwei He

**Affiliations:** ^1^School of Acupuncture and Massage, Jiangxi University of Chinese Medicine, Nanchang, China; ^2^Department of Acupuncture and Moxibustion, The Affiliated Hospital of Jiangxi University of Chinese Medicine, Nanchang, China

**Keywords:** headache, acupuncture treatment, CiteSpace, VOSviewer, Bibliometrix, bibliometric analysis, research trends

## Abstract

**Background:**

While acupuncture treatment has gained extensive usage in addressing headaches, there remains a notable gap in the literature analysis for this field. Therefore, this study aims to conduct a literature review using Citespace, VOSviewer, and Bibliometrix, aiming to examine the current status, strengths, and potential future directions in the utilization of acupuncture for headache treatment.

**Methods:**

Relevant literature on acupuncture treatment for headaches between 2003 and 2023 was retrieved from the Web of Science (WoS) core database. Utilizing CiteSpace 6.1.R6, VOSviewer 1.6.18, and Bibliometrix 4.1.4, we conducted bibliometric analyses across various categories, including countries/regions, institutions, authors, journals, references, and keywords.

**Results:**

A total of 808 research reports were included. China and the United States have significantly contributed to this field. Chengdu University of Chinese Medicine holds the record for the highest number of published papers. Liu Lu has the highest publication output, while Linde K has the highest citation rate. *MEDICINE* leads in publication frequency, while *CEPHALALGIA* holds the highest citation rate. The Long-term Effect of Acupuncture for Migraine Prophylaxis a Randomized Clinical Trial is the most cited reference. Migraine was the most researched type. Filiform needle acupuncture was the most widely used stimulation method. The safety and efficacy of acupuncture have received significant attention. Modern mechanism research shows that depression, brain functional connectivity, and neuroimaging technology have become research hotspots in the acupuncture treatment of headaches.

**Conclusion:**

Acupuncture treatment for headaches has established a stable trend with a promising developmental trajectory. Research in this field mainly focuses on different acupuncture prevention and treatment for various types of headaches, the safety and efficacy of acupuncture, etc. Research on the mechanism of action mainly focuses on interpreting bidirectional and holistic regulation between pain and emotion by acupuncture and the regulation of brain function connection and neuroimaging technology by acupuncture. Future research should expand on the advantages and indications of acupuncture treatment for different headaches and their modern mechanisms.

## 1 Introduction

Headache refers to the sensation of pain or discomfort experienced in the cranial region of an individual and is one of the most common health issues (Steiner et al., 2019; Robbins, 2021). Epidemiological research indicates that a significant portion of the global population has experienced headaches, with the occurrence rate showing notable geographical and demographic disparities (Stovner et al., 2007). The International Headache Society categorizes headaches into primary and secondary divisions. Primary headaches occur independently as diseases or symptoms, such as migraines, tension-type headaches, and cluster headaches, each with distinct clinical features and pathogenic mechanisms (IHS, 2018). Other illnesses or conditions, including elevated intracranial pressure, head trauma, infections, tumors, and cervical spine disorders, cause secondary headaches. Regardless of the type of headache, recurrent episodes can significantly impact patients’ quality of life and work efficiency and even induce psychological and emotional health issues (GBD 2015 Neurological Disorders Collaborator Group, 2017; Lin et al., 2020; Simic et al., 2020).

In modern medicine, treatment methods for headaches can be primarily categorized into non-pharmacological and pharmacological treatments (Freitag and Schloemer, 2014). Non-pharmacological treatment encompasses lifestyle modifications, dietary adjustments, psychological therapy, physical therapy, etc., whereas pharmacological treatment involves the administration of non-steroidal anti-inflammatory drugs, tricyclic antidepressants, analgesics, etc. (Martelletti et al., 2007; Smitherman et al., 2011; Licina et al., 2023). Studies indicate that the efficacy of either non-pharmacological or pharmacological treatment alone is limited (Grazzi et al., 2007). Combining these two approaches can significantly decrease headache-related parameters and widespread pressure pain sensitivity, effectively reducing headaches’ frequency and severity (Deodato et al., 2022). In summary, the concurrent utilization of non-pharmacological and pharmacological treatments is deemed a promising therapeutic strategy.

In this context, acupuncture, as a non-pharmacological therapy, is increasingly garnering attention from scholars. Acupuncture is considered to have a specific efficacy in headache management (Cady and Farmer, 2015). Acupuncture can alleviate headache symptoms, including migraines, tension-type headaches, and headaches caused by other factors (Shih et al., 2017; Dong et al., 2019; Fan et al., 2022; Huang et al., 2022; Li et al., 2022). Furthermore, some researchers have explored the mechanism of action of acupuncture treatment for headaches, suggesting that acupuncture can play an analgesic role by regulating nervous system activity, influencing pain signal transmission, promoting the release of endogenous substances such as endorphins, regulating inflammatory responses, and affecting neural plasticity (Chen et al., 2022). These findings provide theoretical support for research on acupuncture in treating headaches.

In recent years, the field of acupuncture treatment for headaches has gradually received broader attention from the academic community, accompanied by a substantial accumulation of research findings. Although this trend has enriched our knowledge base, it has also presented a challenge: researchers find it difficult to quickly grasp the latest outcomes of current studies and future trends. Given this, there is an urgent need for an innovative method to organize and analyze this massive amount of research literature (Rodrigues et al., 2014). Bibliometrics has emerged in response, relying on bibliometric mapping technology to conduct quantitative analysis of the bibliographic characteristics of literature, revealing the research production patterns of specific fields and visualizing the structure and development trends of research literature (Kokol et al., 2021).

Although existing studies have explored issues such as migraines, tension headaches, and neuropathic pain through bibliometric analysis, bibliometric research on acupuncture treatment for headaches is relatively lacking. This indicates that there is room and a need for further exploration and in-depth research in this field. This study plans to retrieve English literature on acupuncture treatment for headaches published in the Web of Science (WoS) core database from 2003 to 2023. We will use software tools such as CiteSpace, VOSviewer, and Bibliometrix to conduct a detailed analysis of aspects such as countries/regions, institutions, authors, journals, references, keyword co-occurrence, time zone graph, clustering, and burst words, aiming to explore the current status, hot issues, strengths, and future trends of research on acupuncture treatment for headaches.

## 2 Methods

### 2.1 Search strategy

This study extracted literature from the Science Citation Index Expanded database within the WoS core collection via the Jiangxi University of Chinese Medicine library website. The search formula was TS = (“Acupuncture” OR “Pharmacoacupuncture Treatment” OR “autotomy” OR “Electroacupuncture” OR “Electro-acupuncture” OR “Body Acupuncture” OR “Manual Acupuncture” OR “Auricular” OR “Auricular Acupuncture” OR “Auricular Needle” OR “Acupuncture Point” OR “Ear Acupuncture” OR “Warm Acupuncture” OR “needle warming moxibustion” OR “Moxibustion” OR “moxibustion” OR “Thermal moxibustion” OR “Acupoint Injection” OR “Catgut Embedding” OR “Catgut Implantation at Acupoint” OR “Embedding Thread” OR “Dry needle” OR “transcutaneous electrical acupoint stimulation” OR “Scalp acupuncture” OR “fire needle”) AND TS = (“primary headache” OR “tension headache” OR “migraine” OR “cluster headache” OR “cervicogenic headache” OR “trigeminal autonomic cephalalgias” OR “Cephalalgia” OR “headache”), and the search period extended from January 1, 2003, to December 31, 2023. The selected literature types included “Article” and “Reviewer,” while the language type was limited to “English.” The less representative record types, such as editorial materials, meeting abstracts, letters, and other documents etc., were filtered out.

### 2.2 Data processing

**Figure 1:**
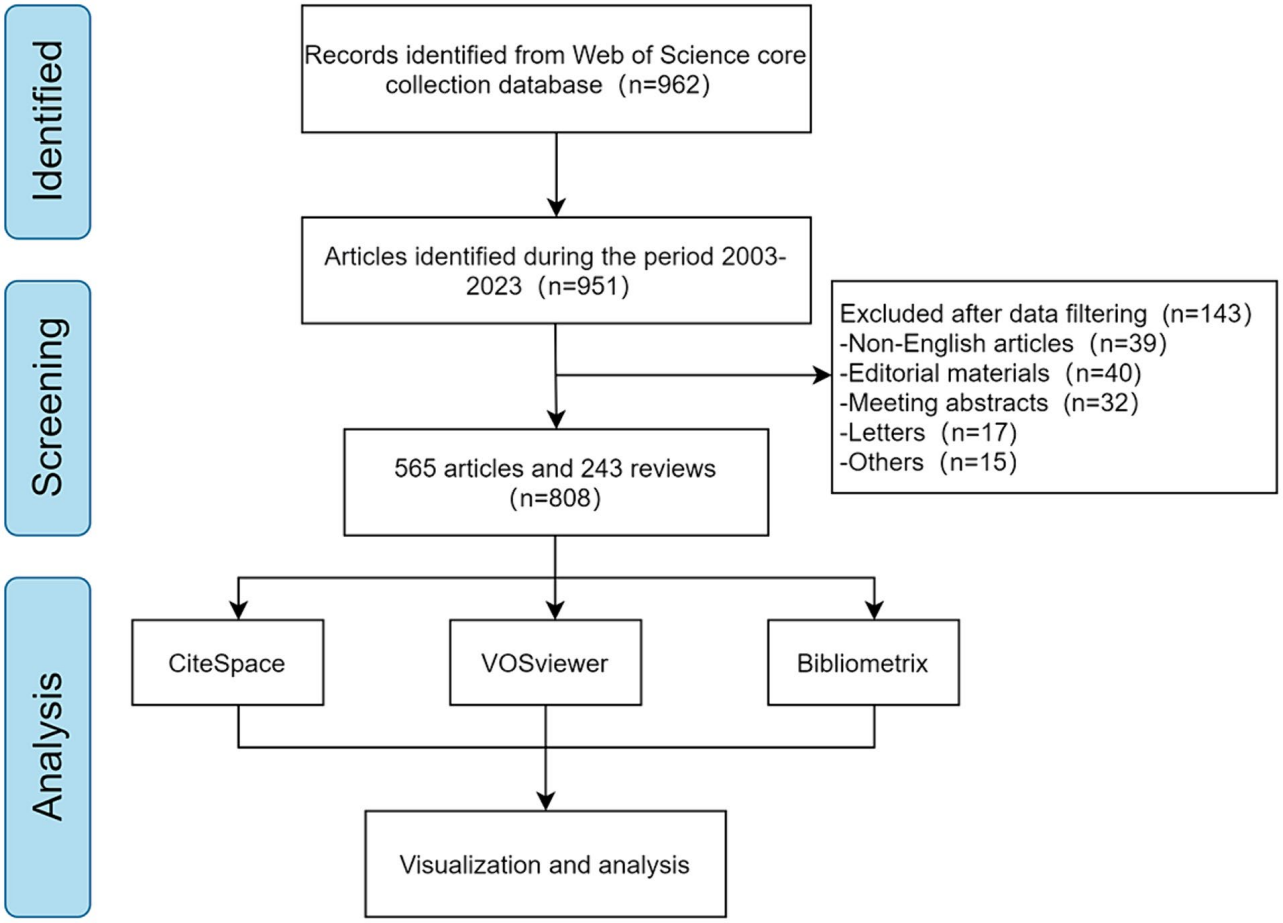
Flow chart of literature screening.

A total of 808 articles were gathered through this search. These literature records were downloaded and saved as plain text files in the “Full Record and Cited References” format. The data collection flow chart is shown in Figure 1.

### 2.3 Data analysis

This study imported text files into CiteSpace 6.1.R6, VOSviewer 1.6.18, and Bibliometrix 4.1.4 for further bibliometric analysis and visualization. The software was used to extract data for analysis, such as countries/regions, institutions, authors, journals, references, keywords, and key metrics for measuring research performance [publication count, centrality, proportion, total citations, average citations, H-index, impact factor (IF), and Journal Citation Reports (JCR)].

CiteSpace is a tool for visualizing citations and conducting analysis, which has gained prominence in scientific measurement and data visualization (Chen, 2006). In this study, CiteSpace was used to analyze countries/regions, journals, references, keywords, and the software parameters are set as follows: (1) Time slicing: 2003–2023, years per slice; (2) Selecting Node Types: All node types were chosen for each analysis; (3) Selection Criterion: g-index: *k* = 25; (4) Pruning: Pathfinder, Pruning sliced networks and Pruning merged network. The visualization employs concentric tree rings (nodes) and connecting lines to depict units and their collaborations. Bigger rings denote increased unit activity, while thicker lines indicate greater collaboration. The color scheme indicates a temporal shift, with cool to warm tones representing the transition from the past to the present. Red nodes signify frequent bursts, and pink-bordered rings highlight the central nodes’ significance (Chen, 2005).

VOSviewer, developed by the Centre for Science and Technology Studies at Leiden University in the Netherlands, is a software designed to create bibliometric networks for visualizing scientific literature (van Eck and Waltman, 2010). This research utilizes VOSviewer to construct networks, focusing on identifying key scholars in the field of acupuncture treatment for headaches through author collaboration and co-citation analysis. In the visualized maps, each node represents a different research element, and the lines between nodes indicate their relationships, with these connections weighted according to their total link strength. Moreover, the spatial distance between nodes reflects the closeness of their relationships.

Bibliometrix is a comprehensive bibliometric analysis toolkit developed for the R language, which assists users in exploring and understanding various metrics and development trends in scientific research (Aria and Cuccurullo, 2017). This study employs Bibliometrix to create keyword time zone graphs, tracking high-frequency keywords and their development trends.

## 3 Results

### 3.1 Analysis of annual publications

**Figure 2:**
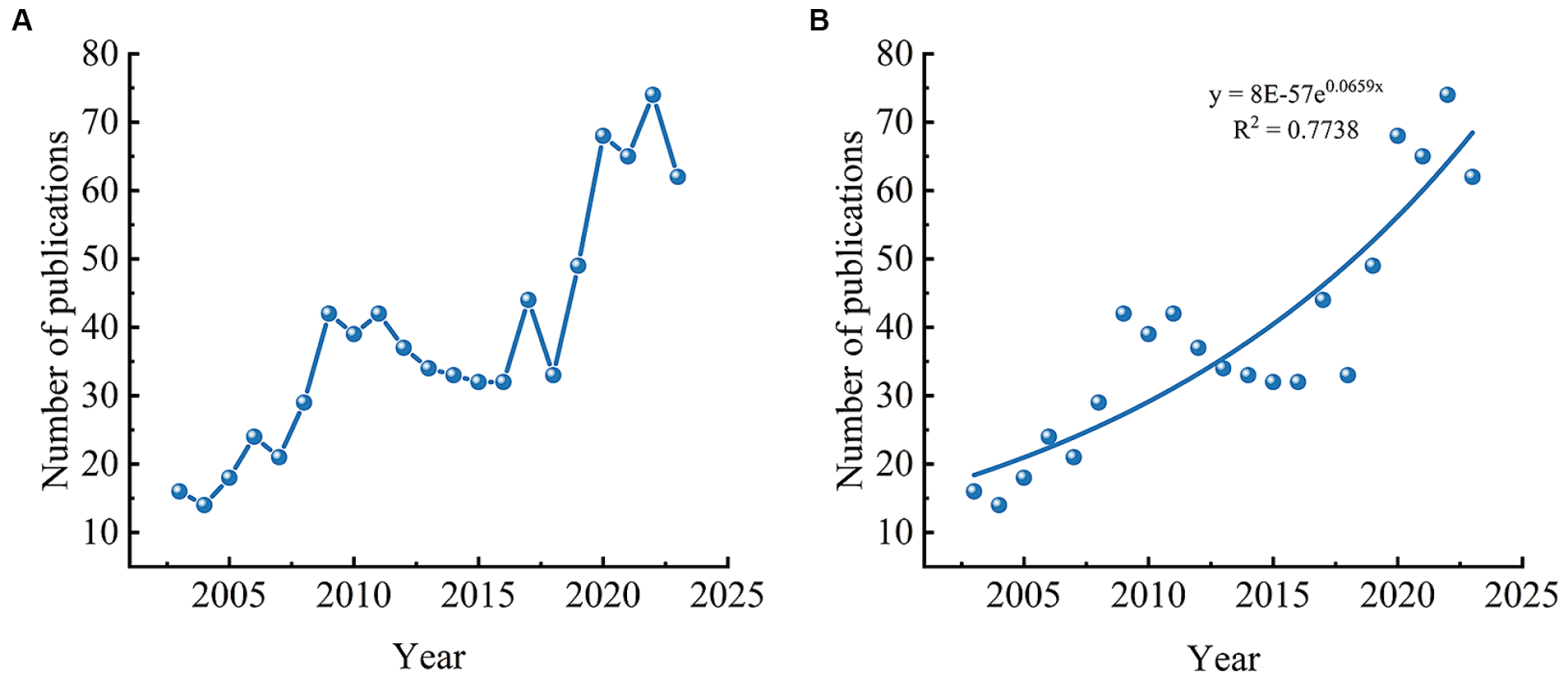
**(A)** The annual number of publications related to acupuncture treatment on headache. **(B)** Model fitting curves of growth trends and prediction of publications numbers in the future.

A total of 808 relevant literature pieces on acupuncture treatment for headaches were included. The publication volume is depicted in Figure 2A. The publication volume can be roughly divided into three stages: the first stage, from 2003 to 2008, represents a period of slow development. During this period, the publication volume fluctuated but gradually grew, averaging 20 articles per year. The second stage, from 2009 to 2017, denotes a stable phase. The publication volume remained relatively constant, showing a slight decline over increasing years, averaging 37 articles per year. The third stage, from 2018 to 2023, signifies rapid growth. Compared to the first stage, the publication volume increased rapidly, averaging 59 articles per year. This indicates a robust growth trend in recent years for research on acupuncture treatment for headaches. Fit the curve in conjunction with the publication’s growth model (Figure 2B); the closer *R*^2^ is to 1, the better the fit, *R*^2^ = 0.7738; it is predicted that the output rate of publications will increase significantly in the future.

### 3.2 Analysis of countries/regions

**Figure 3:**
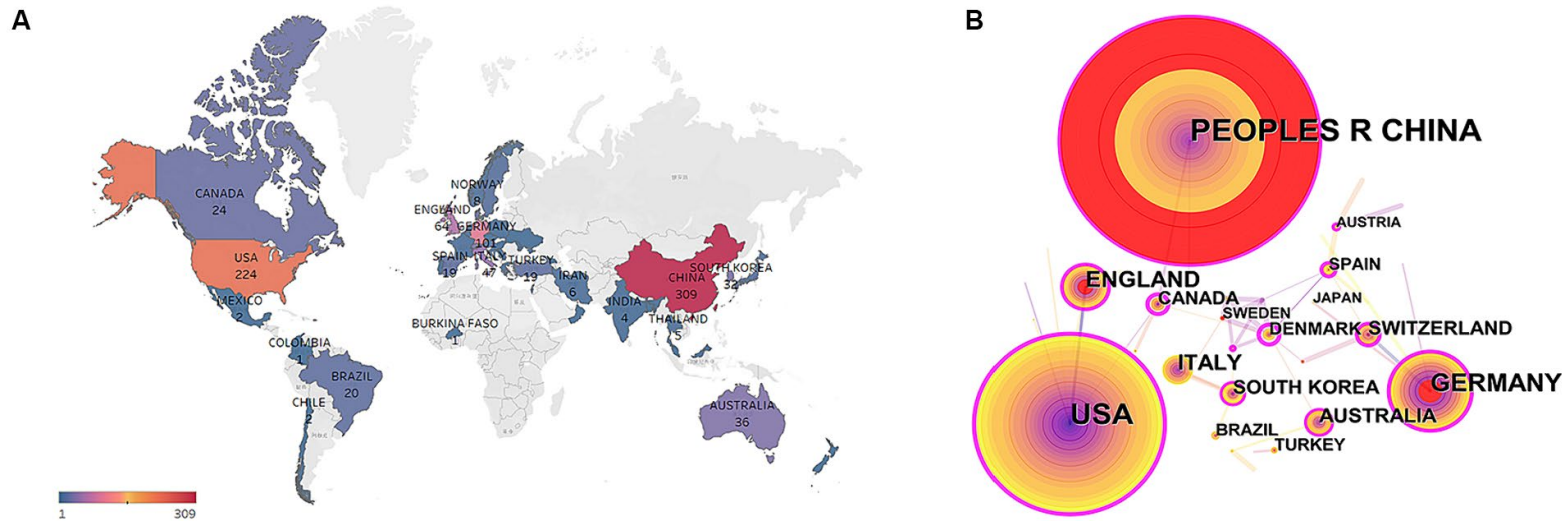
**(A)** World map based on the total publications of different countries/regions. **(B)** Network map of co-authorship between countries/regions related to acupuncture treatment on headache.

Over the past 21 years, research efforts have been conducted in 45 countries/regions (Table 1). China, located in Asia, holds the first position with 309 articles, followed by the United States with 224 pieces. China and the United States are recognized as the central forces driving research in this field, accounting for 65.9%. Other countries have also made contributions, such as Germany (*n* = 101, 12.5%), England (*n* = 64, 7.9%), Italy (*n* = 47, 5.8%). Furthermore, since 2003, reports from 28 countries/regions (accounting for 62.22% of the total) have amounted to less than 10, indicating that most countries/regions are still relatively unexplored in this domain. Figure 3A shows the global distribution of publications in this field.

**Table 1:** The top 5 countries/regions related to acupuncture treatment on headache.

Rank	Country/regions	Counts	Centrality	Proportion (%)
1	China	309	0.26	38.2
2	United States	224	0.33	27.7
3	Germany	101	0.22	12.5
4	England	64	0.22	7.9
5	Italy	47	0	5.8

By selecting nodes within CiteSpace, a map of countries/regions was generated (Figure 3B). Node size corresponds to the total publication count, and lines between nodes represent collaborative relationships. The analysis indicates that China and the United States are pivotal players in research within this field, each establishing their distinct international collaborative networks. However, the lack of connecting lines between the two countries suggests a lack of effective international exchange and collaboration. In terms of national centrality, the top three ranks belong to the United States (0.33), China (0.26), Germany (0.22), and England (0.22). The red nodes representing China, England, and Germany indicate a potential increase in the number of papers published by these countries during a specific period.

### 3.3 Analysis of institutions

**Figure 4:**
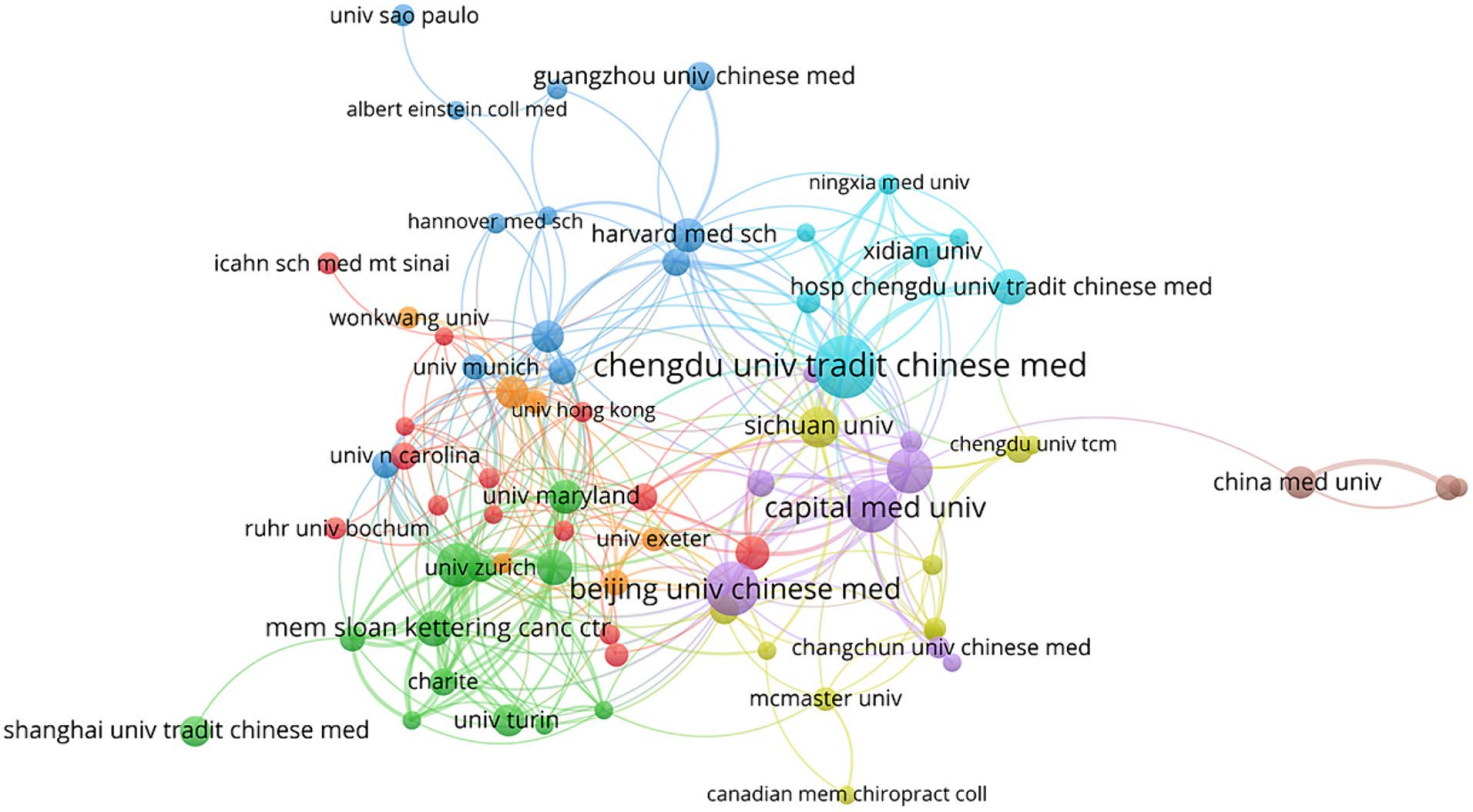
Network map of co-authorship between institutions related to acupuncture treatment on headache.

A total of 482 institutions participated in the research on acupuncture treatment for headaches (Figure 4). The top five institutions with the most published articles are listed in Table 2; among these, Chengdu University of Traditional Chinese Medicine ranks first with 64 papers, accounting for 7.9%, showcasing its robust research capabilities within this field. Following this, other prominent institutions include Beijing University of Chinese Medicine (*n* = 45, 5.6%), Capital Medical University (*n* = 42, 5.2%), China Academy of Chinese Medical Sciences (*n* = 31, 3.8%), and Harvard University (*n* = 29, 3.6%). The two institutions with the highest centrality are the China Academy of Chinese Medical Sciences (0.14) and Harvard University (0.12). It is worth noting that although China has the advantage in the number of publications, Harvard University in the United States has the highest total citations (2127), average citations (73.34), and H-index (24), showing a significant influence in this field. This information reflects the critical contributions of China and the United States to global acupuncture and headache research.

**Table 2:** The top 5 institutions related to acupuncture treatment on headache.

Rank	Institution	Counts	Centrality	Proportion (%)	Total citations	Average citations	*H*-index
1	Chengdu Univ Tradit Chinese Med	64	0.09	7.9	1,507	23.55	20
2	Beijing Univ Chinese Med	45	0.06	5.6	877	19,49	16
3	Capital Med Univ	42	0.04	5.2	655	15.60	15
4	China Acad Chinese Med Sci	31	0.14	3.8	366	11.81	11
5	Harvard Univ	29	0.12	3.6	2,127	73.34	24

### 3.4 Analysis of authors and co-cited-authors

Author network map identifies prominent authors in the field and quantifies the extent of their collaboration. Node size corresponds to the number of publications among authors. As indicated in Table 3, the highest publication count for acupuncture treatment of headaches belongs to Liu Lu from the Acupuncture Department of Beijing Traditional Chinese Medicine Hospital, with a total of 21 articles (2.6%). The most highly cited article authored by Liu Lu is “Effect of electroacupuncture pretreatment at GB20 on behavior and the descending pain modulatory system in a rat model of migraine,” which demonstrates that electroacupuncture preconditioning can improve behavioral changes in a rat model of recurrent migraine, possibly through the regulation of the brainstem descending pathway (Pei et al., 2016). The second-ranked author is Zhao Ling from Chengdu University of Traditional Chinese Medicine, with 19 articles (2.4%). The most highly cited article authored by Zhao Ling is “The Long-term Effect of Acupuncture for Migraine Prophylaxis A Randomized Clinical Trial,” which explores the long-term efficacy of true acupuncture (TA) versus sham acupuncture (SA) in migraine prophylaxis. The conclusion suggests that TA might be associated with long-term reduction in migraine recurrence among patients without aura (Zhao et al., 2017). The third-ranked author, Liang Fanrong, participated in the writing of the articles above. Other authors include Linde, K., Witt, C. M., Allais, G., and Macpherson, H. Among these, Linde, K has the highest total citations (4020), average citations (236.47) and H-index(24). These authors have conducted research from various perspectives, such as the impact of chronic pain patients’ expectations on acupuncture efficacy (Linde et al., 2007), meta-analysis of chronic pain patient data (Vickers et al., 2012), the effectiveness of acupuncture for migraine prophylaxis (Linde et al., 2009) and revising acupuncture clinical trial intervention reporting standards (MacPherson et al., 2010), laying the foundation for subsequent studies.

**Table 3:** Authors with more than 10 publications related to acupuncture treatment on headache.

Rank	Author	Counts	Proportion (%)	Total citations	Average citations	*H*-index	Magnum opus (publication year)
1	Liu, Lu	21	2.6	166	7.90	9	Effect of electroacupuncture pretreatment at GB20 on behavior and the descending pain modulatory system in a rat model of migraine (2016)
2	Zhao, Ling	19	2.4	492	25.89	8	The Long-term Effect of Acupuncture for Migraine Prophylaxis A Randomized Clinical Trial (2017)
3	Liang, Fanrong	17	2.1	1,233	72.53	16	
4	Linde, K	17	2.1	4,020	236.47	24	The impact of patient expectations on outcomes in four randomized controlled trials of acupuncture in patients with chronic pain (2007)
5	Witt, C. M.	14	1.7	3,003	214.50	17	Acupuncture for Chronic Pain Individual Patient Data Meta-analysis (2012)
6	Allais, G	13	1.6	882	67.85	12	Acupuncture for migraine prophylaxis (2009)
7	Macpherson, H	11	1.4	2,420	220	15	Revised Standards for Reporting Interventions in Clinical Trials of Acupuncture (STRICTA): Extending the CONSORT Statement (2010)

**Figure 5:**
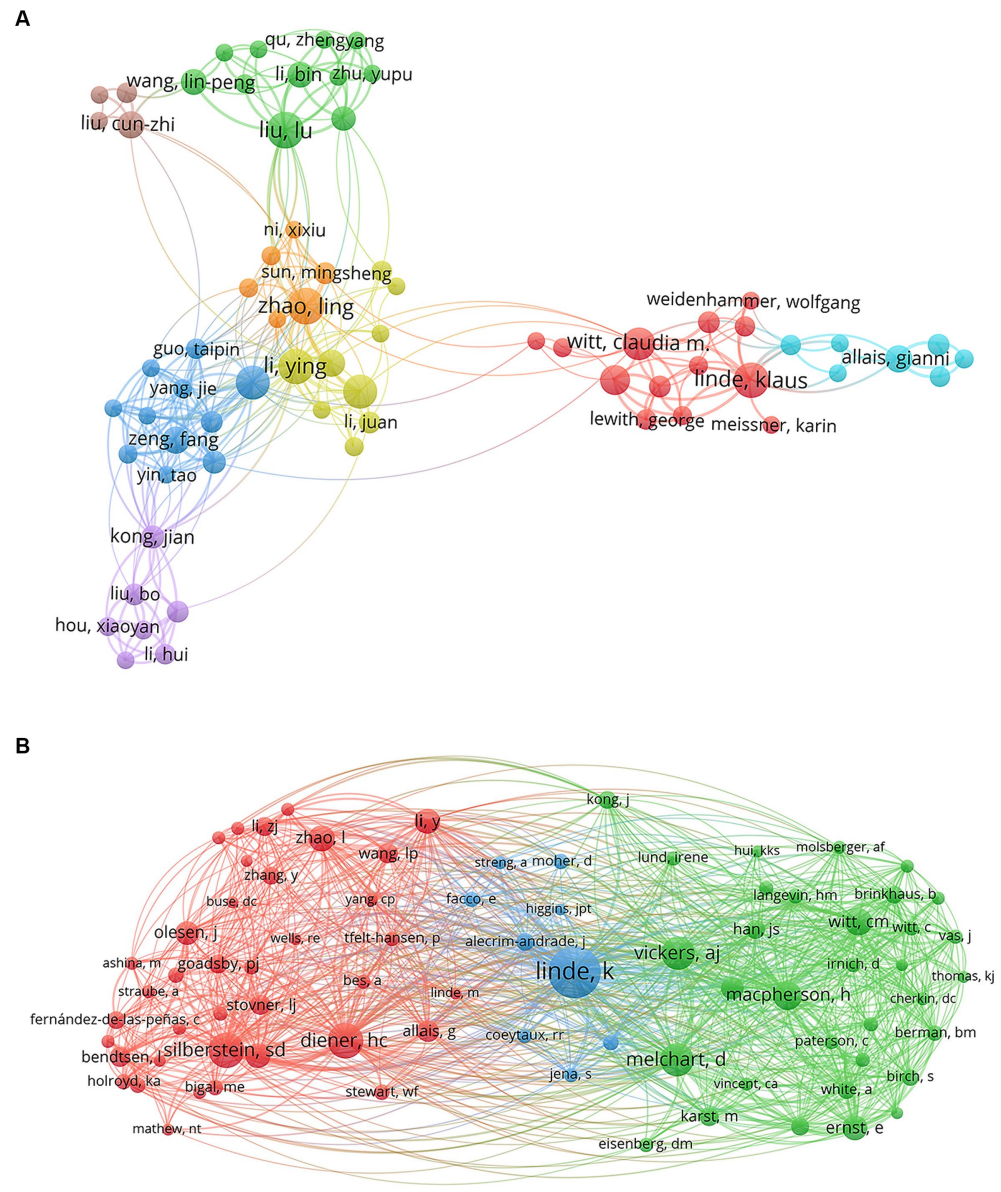
**(A)** Network map of co-authorship between authors related to acupuncture treatment on headache. **(B)** Network map of co-cited authors related to acupuncture treatment on headache.

From the author network map in Figure 5A, it is evident that Witt, C. M. serves as an important hub for Western-Eastern academic exchange. Additionally, authors with a significant publication volume have formed stable research teams within the same research institutions. The team led by Liu Lu, Li Bin, and Wang Linpeng from Capital Medical University primarily focuses on using a rat model of migraine to study the underlying mechanisms of EA’s effect on improving migraines. The team consisting of Zhao Ling, Liang Fanrong, and Li Ying from Chengdu University of Traditional Chinese Medicine has extensively researched acupuncture’s prevention of clinical trial headaches, its long-term efficacy, and safety. The team led by Linde, K and Allais, G from the Technical University of Munich is dedicated to investigating whether there are differences in effectiveness between true acupuncture and sham acupuncture.

Of the 191 co-cited authors (Figure 5B), 13 have received citations exceeding 100 times. Table 4 displays the top five co-cited authors and centrality. Linde K from Germany led the list with 557 citations, and Lipton, RB from the USA ranked first with a centrality of 0.35. The 10 co-cited authors are affiliated with Germany and the United States, underscoring the pivotal contribution of publications from these countries in advancing further research on acupuncture’s efficacy in treating headaches.

**Table 4:** Top 5 co-cited authors and centrality related to acupuncture treatment on headache.

Rank	Citations	Co-cited author	Country	Rank	Centrality	Co-cited author	Country
1	557	Linde, K	Germany	1	0.35	Lipton, RB	United States
2	253	Melchart, D	Germany	2	0.3	Gaul, C	Germany
3	195	Diener, HC	Germany	3	0.21	Pfaffenrath, V	Germany
4	168	Vickers, AJ	United States	4	0.18	Eisenberg, DM	United States
5	166	Silberstein, SD	United States	5	0.18	Hesse, J	Germany

### 3.5 Analysis of journals and cited journals

In a combined total, 808 papers were published across 200 journals. Table 5 presents the top five journals with the highest popularity; the journal with the most publications was *MEDICINE* (*n* = 36, 4.5%), followed by *EVID-BASED COMPL ALT* (*n* = 34, 4.3%), *ACUPUNCT MED* (*n* = 31, 3.9%), *J ALTERN COMPLEM MED* (*n* = 31, 3.9%), and *TRIALS* (*n* = 29, 3.6%). Unfortunately, these journals typically have an impact factor (IF) of less than five and rank in the Q3 and Q4 categories.

**Table 5:** Top 5 journals related to acupuncture treatment on headache.

Rank	Journal	Counts	Proportion (%)	Total citations	Average citations	IF (2022)	JCR (2022)
1	MEDICINE	36	4.5	149	4.14	1.6	Q4
2	EVID-BASED COMPL ALT	34	4.3	696	20.47	0	Q3
3	ACUPUNCT MED	31	3.9	651	21	2.5	Q3
4	J ALTERN COMPLEM MED	31	3.9	929	29.97	2.6	Q3
5	TRIALS	29	3.6	429	14.79	2.5	Q3

**Figure 6:**
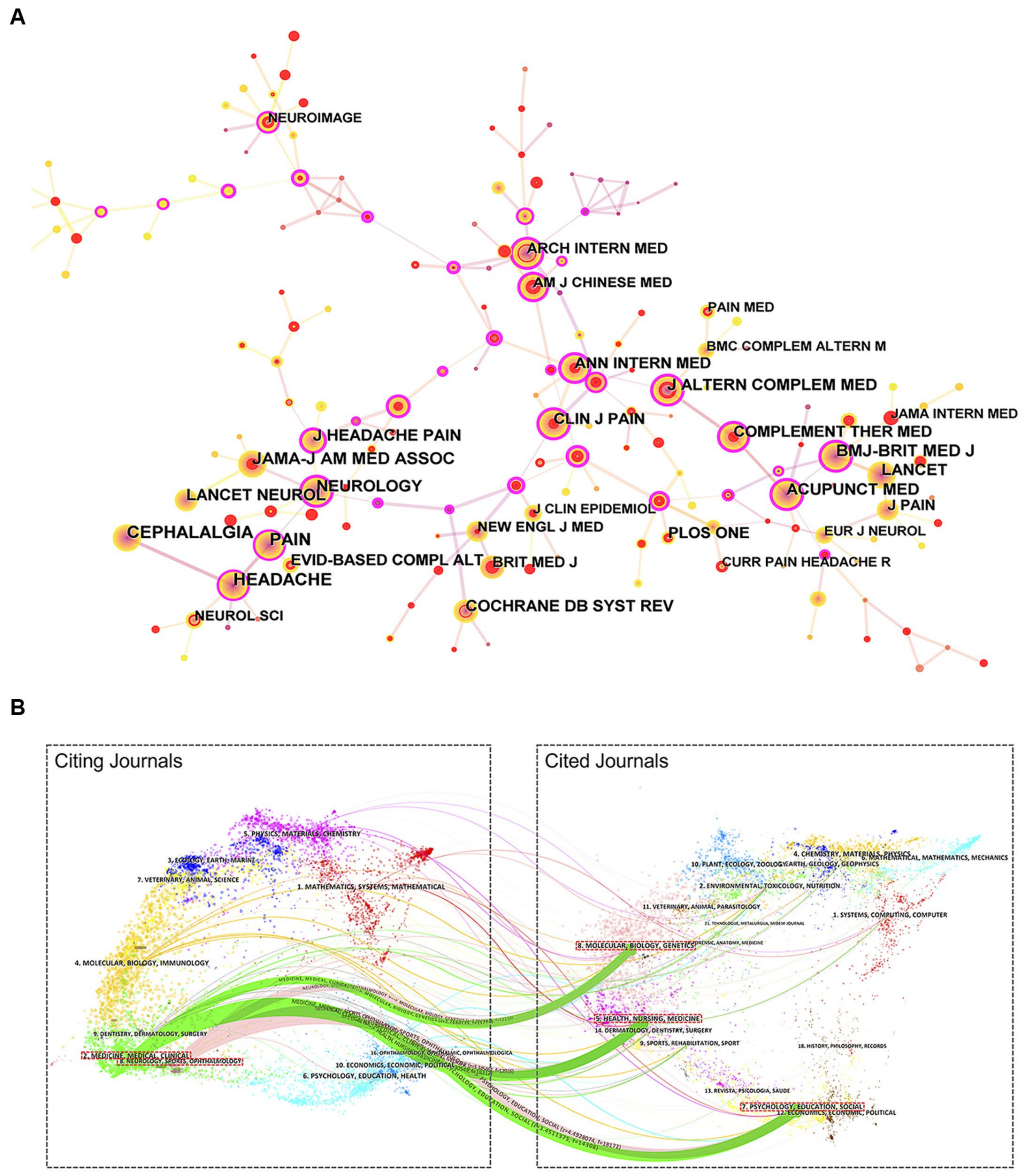
**(A)** Network map of co-cited journals related to acupuncture treatment on headache. **(B)** Dual-map overlay of journals related to acupuncture treatment on headache.

Table 6 displays the top five academic journals with more than 300 citations. *CEPHALALGIA* holds the top position with 502 citations, trailed by *HEADACHE* (466), *PAIN* (444), *JAMA-J AM MED ASSOC* (372), and *BMJ-BRIT MED J* (334). These cited journals are all classified as Q1 and Q2. *J AMA-JAM MED ASSOC* has the highest total citations (1772693), average citations (55.25), and IF (120.7). Figure 6A shows a network map of more than 100 cited journals.

**Table 6:** Top 5 co-cited journals related to acupuncture treatment on headache.

Rank	Co-cited journal	Citations	Centrality	Total citations	Average citations	IF (2022)	JCR (2022)
1	CEPHALALGIA	502	0.09	123,539	10.78	4.9	Q1
2	HEADACHE	466	0.10	97,737	12	5	Q2
3	PAIN	444	0.12	399,755	52.99	7.4	Q1
4	JAMA-J AM MED ASSOC	372	0.10	1,772,693	55.25	120.7	Q1
5	BMJ-BRIT MED J	334	0.06	1,008,929	23.71	105.7	Q1

Figure 6B is a dual-map overlay of the journal and the cited journal. The left side of the dual map is citing journals while the right side is cited journals, and the line in the middle indicates the association between them, showing the co-occurrence network regarding acupuncture treatment for depression. There were five major citation paths on the current map. The citing matrices of journals focused on two main areas: (1) medicine, medical, and clinical, and (2) neurology, sports, and ophthalmology, whereas the most cited publications originated from the journals in the field of (1) molecular biology and genetics, (2) health, nursing, and medicine, and (3) psychology, education, and social.

### 3.6 Analysis of references

**Figure 7:**
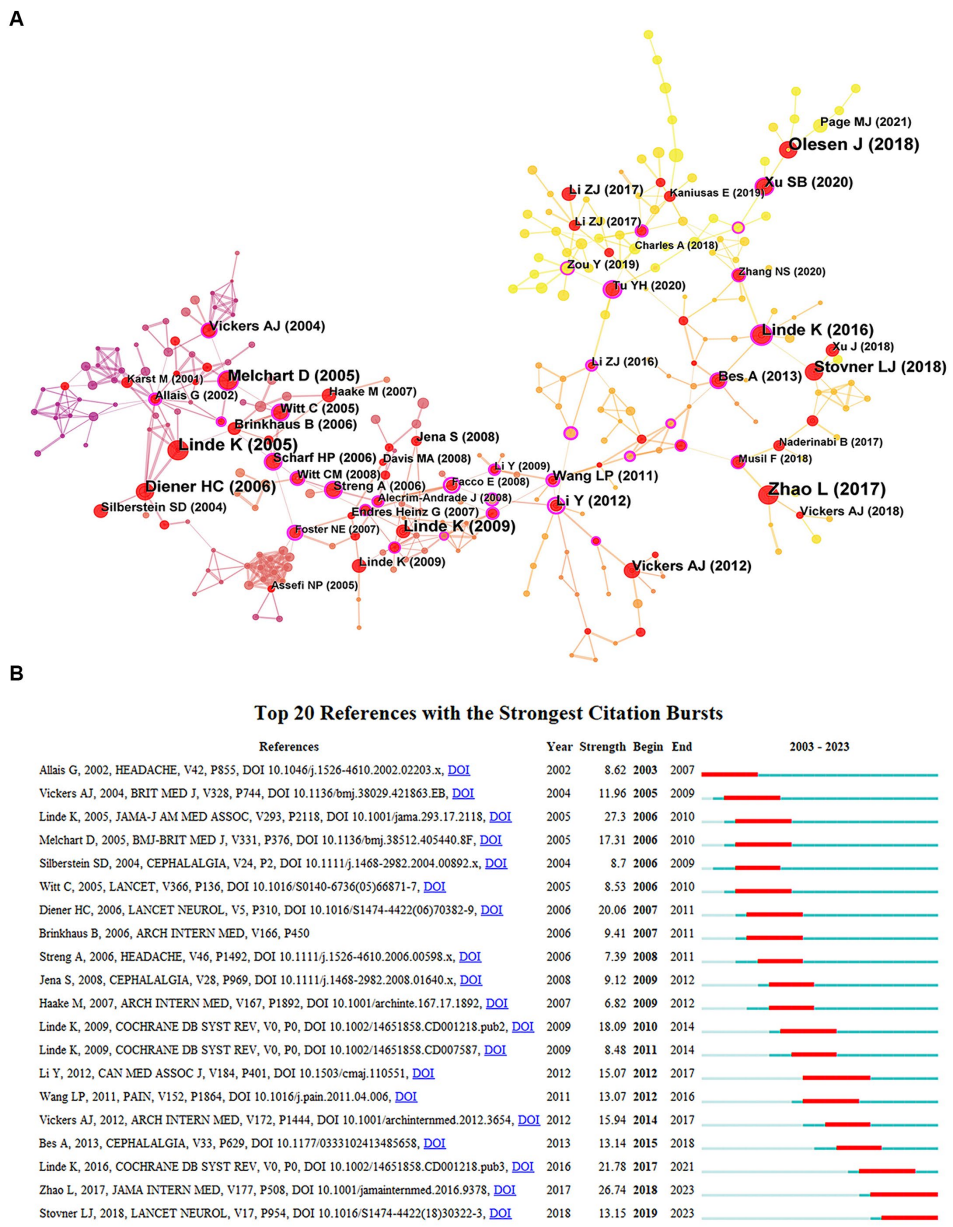
**(A)** Network map of references related to acupuncture treatment on headache. **(B)** The top 20 references with the strongest citation bursts related to acupuncture treatment on headache.

Table 7 lists the five research papers with the highest citation frequencies, with Zhao (2017), Olesen (2018), and Linde (2005) ranking in the top three. Notably, Linde K and his team published two groundbreaking articles in 2016 and 2019, primarily discussing the effectiveness of acupuncture treatment for migraines. Figure 7A shows a close relationship between references with a higher number of citations, further corroborating that high-quality research papers are inseparable from the support of excellent academic works. We analyzed the strong citation bursts in the literature using CiteSpace, identifying 20 documents of significant impact. As shown in Figure 7B, light blue stripes indicate that the keyword has not yet appeared, dark blue stripes indicate that the keyword has occurred, and red stripes indicate the period when the keyword has experienced a burst. The most cited documents in the past 2 years are “The Long-term Effect of Acupuncture for Migraine Prophylaxis: A Randomized Clinical Trial” published in *JAMA INTERN MED* and “Global, regional, and national burden of migraine and tension-type headache, 1990–2016: a systematic analysis for the Global Burden of Disease Study 2016” published in *LANCET NEUROL* (Zhao et al., 2017; Stovner et al., 2018).

**Table 7:** Top 5 references related to acupuncture treatment on headache.

Rank	Citations	Cited reference	Journals	Representative author, publication year
1	74	The long-term effect of acupuncture for migraine prophylaxis a randomized clinical trial	JAMA INTERN MED	Zhao et al. (2017)
2	68	Headache classification committee of the international headache society (IHS) the international classification of headache disorders, 3rd edition	CEPHALALGIA	Olesen (2018)
3	59	Acupuncture for patients with migraine: a randomized controlled trial	JAMA-J AM MED ASSOC	Linde et al. (2005)
4	47	Acupuncture for the prevention of episodic migraine	COCHRANE DB SYST REV	Linde et al. (2016)
5	46	Efficacy of acupuncture for the prophylaxis of migraine: a multicenter randomized controlled clinical trial	LANCET NEUROL	Diener et al. (2006)

### 3.7 Analysis of keywords

Keywords encapsulate the research domain of this paper, and analyzing these keywords aids in identifying current research trends.

#### 3.7.1 Co-occurrence network of keywords

**Figure 8:**
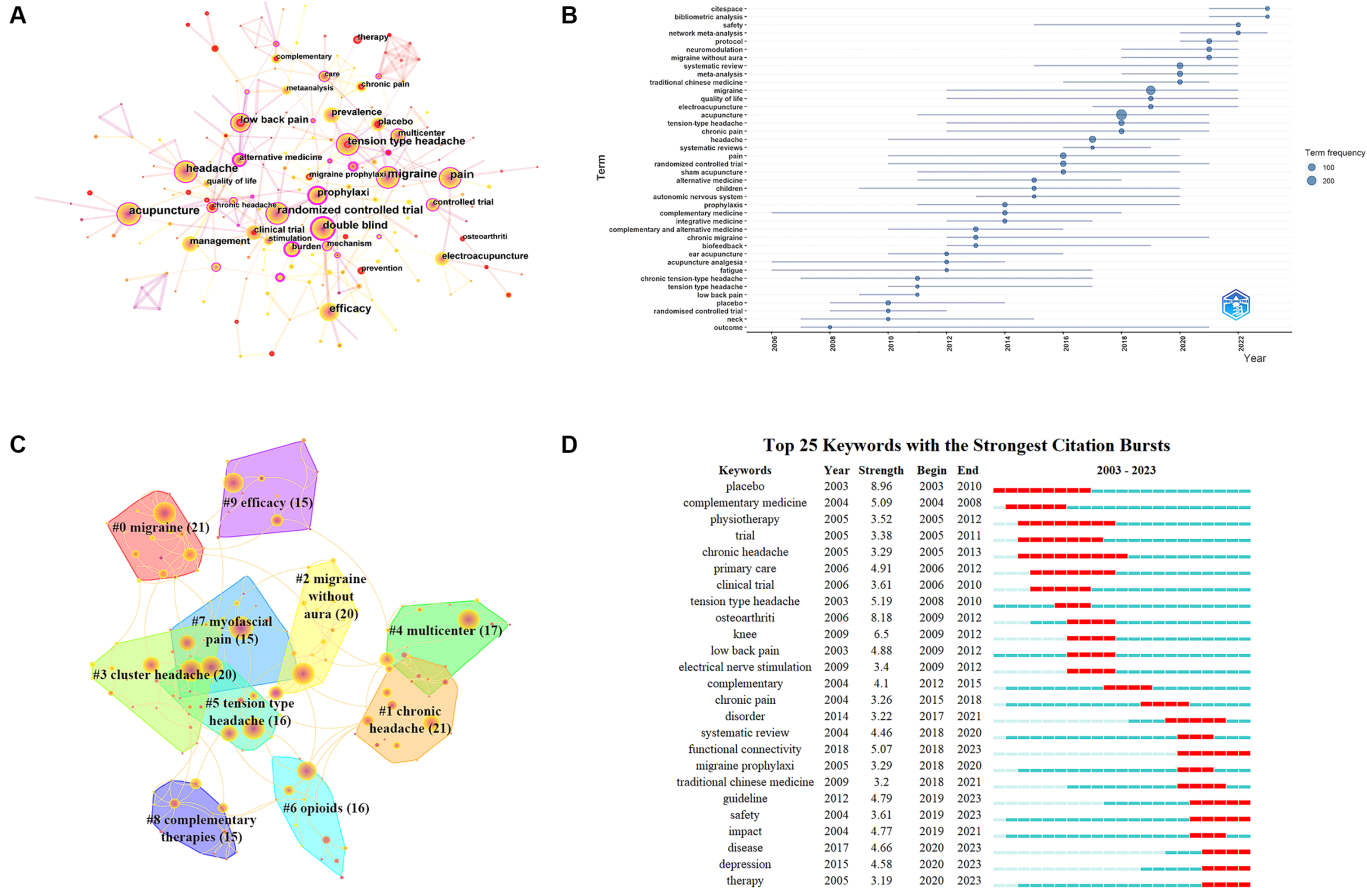
**(A)** Network map of keywords related to acupuncture treatment on headache. **(B)** Keyword time zone related to acupuncture treatment on headache. **(C)** Cluster map of keywords related to acupuncture treatment on headache. **(D)** The top 25 keywords with the strongest citation bursts related to acupuncture treatment on headache.

Table 8 and Figure 8A show the top 10 keywords with high frequency and centrality. The frequency of “acupuncture,” “migraine,” and “pain” ranked the top three, and the centrality of “acupuncture therapy,” “tension type headache,” and “acupuncture analgesia” ranked the top three. We use a time zone graph to demonstrate the evolution of keywords over time (Figure 8B). Keywords are positioned on the vertical axis, and time is on the horizontal axis, with the direction pointing to the right. From 2003 to 2023, the changes in high-frequency keywords over time are clearly presented. This study categorizes the prominent keywords in the past 21 years based on headache type, acupuncture stimulation type, safety, mechanism of action, disease impact, and research methods, etc., as indicated in Table 9:

**Table 8:** Top 10 keywords and centrality related to acupuncture treatment on headache.

Rank	Counts	Keywords	Rank	Centrality	Keywords
1	162	Acupuncture	1	0.19	Acupuncture therapy
2	159	Migraine	2	0.16	Tension type headache
3	134	Pain	3	0.16	Acupuncture analgesia
4	120	Headache	4	0.15	Randomized controlled trial
5	119	Efficacy	5	0.15	Alternative medicine
6	116	Randomized controlled trial	6	0.14	Stimulation
7	101	Tension type headache	7	0.11	Knee osteoarthritis
8	94	Double blind	8	0.11	Cervicogenic headache
9	88	Prophylaxis	9	0.11	Back pain
10	86	Low back pain	10	0.10	Primary care

**Table 9:** Top 5 keywords of all types related to acupuncture treatment on headache.

Rank	Types of stimulation	Type of headache	Bad incident	Mechanism	Popular attention	Research methods
1	Acupuncture	Migraine	Bleeding/hematoma (6.1%)	Neuromodulation	Efficacy	Randomized controlled trial
2	Electroacupuncture	Tension type headache	Pain (1.7%)	Opioid peptides	Pain	Double blind
3	Sham acupuncture	Chronic headache	Vegetative symptoms (0.7%)	5-HT receptors	Prophylaxis	Clinical trial
4	Auricular acupressure	Cervicogenic headache	Nerve injuries (0.2%)	Neurohormone	Prevalence	Multi-center
5	Acupoint pressure	Cluster headache	Others (0.1%)	Functional connectivity	Quality of life	Placebo

(1) Headache types: The top five ranked headache types are “migraine,” “tension type headache,” “chronic headache,” “cervicogenic headache,” and “cluster headache.” This indicates that primary headaches are the most extensively studied, with migraine being the most researched subtype, aligning with basic epidemiological patterns.

(2) Acupuncture stimulation types: The top five ranked acupuncture stimulation types are “filiform needle acupuncture,” “electroacupuncture,” “sham acupuncture,” “auricular acupuncture,” and “acupoint pressure.” This suggests that research on acupuncture treatment for headaches is most prevalent. Experimental processes often involve blank controls, waiting lists, standardized sham acupuncture, ritualized sham acupuncture, sham acupuncture, sham electroacupuncture, and Western medication. Studies utilizing moxibustion as a stimulation method have yet to be reported.

(3) Safety: Most studies do not report adverse events from acupuncture. However, some research indicates an adverse event rate of approximately 8.6% associated with acupuncture. Common adverse reactions include minor bleeding or hematoma (6.1%), pain (1.7%), and vegetative symptoms (0.7%) (Witt et al., 2009). Adverse reactions resulting from negligence or medical accidents (such as forgotten or broken needles, pneumothorax, and burns after moxibustion) account for around 0.1%. Furthermore, the interpretation of adverse events is subjective. For instance, sensations of soreness, numbness, heaviness, or distention after acupuncture, which clinicians view as indications of therapeutic efficacy, might be considered adverse reactions by patients. Therefore, when administered by adequately trained and experienced practitioners, acupuncture proves to be a highly safe practice, with rare instances of severe adverse events.

(4) Mechanisms of action: The top five mechanisms of acupuncture’s analgesic effects include “neuromodulation,” “opioid peptides,” “5-HT receptors,” “neurohormone,” and “functional connectivity,” highlighting the central role of neural modulation in acupuncture’s pain relief. Acupuncture can modulate neuronal activity, activating or inhibiting the central and peripheral nervous systems, thereby effectively reducing pain perception (Chen et al., 2023). Notably, “opioid peptides” and “5-HT receptors” play critical roles in this process (Lin et al., 2022). Research reveals that the endogenous opioid peptides in the central nervous system are crucial for the analgesic effect of electroacupuncture (EA), with different frequencies of EA releasing different types of neuropeptides. For instance, 2 Hz EA promotes the release of endorphins, enkephalins, and dynorphins, while 100 Hz EA specifically enhances the release of dynorphins (Han, 2004). Additionally, acupuncture-induced release of 5-HT at acupoints contributes to its analgesic effect, with ATP secretion mediated by 5-HT1A receptors possibly being a potential mechanism (Li et al., 2023). Acupuncture can also regulate the neuroendocrine system, inducing analgesia by affecting the secretion and balance of hormones. Studies show that EA increases the content of norepinephrine and the expression of *β*2-adrenergic receptors in inflamed tissues, thus alleviating pain (Shi et al., 2023). The latest research gradually reveals the deep mechanisms of acupuncture in relieving pain, especially its close connection with brain functional connectivity (Otti and Noll-Hussong, 2012). Acupuncture can change brainwave activity patterns and regulate the activity of brain regions related to pain perception and processing, thus affecting pain perception and cognition (Zhang et al., 2020b). In summary, the analgesic mechanism of acupuncture is a complex network regulation process covering multiple biological pathways and mechanisms. These mechanisms may vary depending on the type of pain, chosen acupoints, and individual differences.

(5) Popular attention: Public focus on the disease predominantly centers around terms like “efficacy,” “pain,” “prophylaxis,” “prevalence,” and “quality of life.” These focal points reflect society’s expectations and demands for acupuncture as a treatment method to alleviate and prevent headaches.

(6) Research methods: Keywords such as “randomized controlled trial,” “double-blind,” “clinical trial,” “multicenter,” and “placebo” indicate that most studies on acupuncture for treating headaches employ randomized controlled trials. From the perspective of evidence-based medicine, evidence derived from randomized controlled trials is regarded as the highest level of the evidence pyramid, offering strong support for the clinical utilization of acupuncture in the treatment of headaches (Bhide et al., 2018).

(7) Efficacy: High-quality meta-analysis and systematic reviews indicate that acupuncture is effective for migraine, tension-type headache, chronic pain, and cervicogenic headache, making it a favorable option for headache patients (Sun and Gan, 2008; France et al., 2014; Millstine et al., 2017; Lu et al., 2022; Tao et al., 2023).

#### 3.7.2 Clustered network of keywords

The keywords clusters are shown in Figure 8C. The network contained 208 nodes and 311 links. The largest CC was 98%, indicating a high concentration of major research areas or topics; the Modularity Q was 0.7515 (>0.5), suggesting that the network clusters were well-defined and reasonable, and the Mean Silhouette S was 0.9026 (>0.5), indicating that the clusters demonstrated acceptable homogeneity (Chen, 2006). In this network, more important clustering labels were listed in 10 clusters, including “#0 migraine(21),” “#1 chronic headache(21),” “#2 migraine without aura(20),” “#3 cluster headache (20),” “#4 multicenter(17),” “#5 tension type headache(16),” “#6 opioids(16),” “#7 myofascial pain(15),” “#8 complementary therapies(15),” “#9 efficacy(15),” as depicted with varying colors.

#### 3.7.3 Keyword burst detection network

Utilizing the burst detection algorithm in CiteSpace software, this paper conducts keyword burst detection and analysis for 808 articles from the WoS core database. Synonymous keyword nodes are merged, with a minimum duration of 2 years, state transition value at 1, and state difference ratio at 2. This process identifies the top 25 keywords with the highest burst intensities. The distribution of burst times and durations for the top 25 burst keywords is illustrated in Figure 8D. Light blue stripes indicate that the keyword has not yet appeared, dark blue stripes indicate that the keyword has appeared, and red stripes indicate the time period when the keyword has exploded.

Based on the burst detection results, the keyword with the greatest emergences was “placebo” (8.96). Keywords with burst durations exceeding 5 years include “placebo” (2003–2010), “physiotherapy” (2005–2012), “trial” (2005–2011), “chronic headache” (2005–2013), “primary care” (2006–2012), and “functional connectivity” (2018–2023). This indicates the pivotal roles these aspects have played in acupuncture headache research.

From a time perspective, studies from 2003 to 2018 mainly used clinical trials, and multi-center and large-sample randomized controlled clinical trials showed higher research quality and credibility. The emergence of keywords in 2018–2020 reflects the continual rise of systematic reviews during this period. Systematic reviews aim to synthesize and integrate existing study results to derive the best evidence synthesis, making them a pivotal source of evidence-based medical research. During 2020–2023, the keywords “functional connectivity,” “guideline,” “safety,” “disease,” “depression,” and “therapy” had the highest number of outbreaks.

## 4 Discussion

### 4.1 General information analysis

From the perspective of annual publications, the trend of literature publication from 2003 to 2023 has gone through three stages, with the number of publications from 2003 to 2008 showing a slow growth trend and the number of publications from 2009 to 2017 maintaining the same level. In contrast, there was a rapid increase in publication output between 2018 and 2023. Combining curve fitting of the publication growth model, it is predicted that the future publication output rate will significantly increase.

In the network of national and institutional cooperation, 45 countries/regions and 482 institutions have participated in acupuncture research for headache treatment, and China topped the list. Among the top institutions contributing the most articles, four are based in China, reflecting the connection between acupuncture and its origins in China. Moreover, many countries/regions had minimal or no contribution to this research, indirectly highlighting a need for more emphasis on acupuncture-related headache studies in certain Western countries, which can be attributed to Western scholars’ skepticism toward acupuncture’s efficacy and safety, as well as their limited familiarity with traditional Chinese medical theories. Given that China and the United States are the primary driving forces in this field, there has been limited cooperation and exchange between the two nations. Consequently, establishing a long-term and stable collaborative relationship between them holds great significance in advancing the development of this field.

The top three authors with the highest publication output in acupuncture for headache treatment are Liu Lu from the Acupuncture Department of Beijing Hospital of Traditional Chinese Medicine, Zhao Ling, and Liang Fanrong from Chengdu University of Traditional Chinese Medicine. Combined with co-authorship analysis, it’s evident that authors with high publication output often comprise stable research teams within the same institution. This underscores the importance of forming regular research teams for deepening research in this field.

*MEDICINE* holds the record as the most prolific journal in the field, boasting 36 papers. However, it does not appear in the top five journals ranked by the number of co-citations. This suggests that the journal may be supportive of acupuncture-related research but lacks the widespread recognition and impact necessary for broad dissemination. On the other hand, *CEPHALALGIA* is the most cited journal, positioned in the Q1 category, indicating its high academic status and international influence. The Long-term Effect of Acupuncture for Migraine Prophylaxis a Randomized Clinical Trial is the most cited reference. At the same time, it’s clear that the support for high-quality research papers and excellent academic works is inseparable. Therefore, in order to achieve rapid progress in this field of study, scholars must focus on the quality of their references, enhance the standard of their papers, and commit to publishing their work in high-quality academic journals, with the aim of playing a more effective leading role in the field.

### 4.2 Analysis of research hotspots and trends

Building upon the reference literature and keyword analysis and considering the research status in this field, we have conducted an investigation and provided some insights and suggestions regarding future research hotspots and trends:

#### 4.2.1 Diverse disease exploration, with high emphasis on migraine

Headache, as a non-specific symptom, can be associated with various diseases or pathological states. Based on keyword analysis, the research hotspots in this field primarily centers around the efficacy of acupuncture in preventing and treating migraines. In the past decades, acupuncture has been pointed out as a valuable non-pharmacological tool in patients with migraine. In acupuncture research, true acupuncture is often compared with sham acupuncture. There are many different types of sham acupuncture intervention; these include lack of skin penetration by the needle, shallow penetration of the needle, insertion at points that are not traditional acupuncture points, or not achieving “deqi” which is an expected needling response (subjective sensation of local warmth, paresthesia tenderness) that is considered an integral element of the healing process (Zhang et al., 2020a). The efficacy of acupuncture was also reported in comparison with pharmacological treatment in episodic migraine (Hesse et al., 1994; Allais et al., 2002; Diener et al., 2006; Streng et al., 2006; Wang et al., 2011; Facco et al., 2013; Zhang et al., 2020a). The majority of these studies compared acupuncture with monotherapy as a prophylactic treatment. However, recently, one prospective, randomized controlled, study compared acupuncture with the best prophylactic drugs for patients taking into consideration comorbidities (i.e., depression, insomnia, hypertension, etc.) and previous preventive treatment (Giannini et al., 2021). This trial showed that acupuncture was as effective as the more appropriate pharmacological treatment for migraine prophylaxis. Together, this data, support the efficacy of acupuncture in migraine prophylaxis. Furthermore, the neural mechanisms of acupuncture for migraine treatment have garnered increasing attention. Neuroimaging studies suggest that acupuncture may alter aberrant functional activity and connectivity in the descending pain modulation system, default mode network, thalamus, anterior cingulate cortex, posterior cingulate cortex, and cerebellum. Acupuncture regulates peripheral and central sensitization, normalizes abnormal brain activity, and thus inhibits the transmission of pain signals (Chen et al., 2022). Additionally, tension-type headache has been a focus. The Cochrane systematic review database acknowledges acupuncture’s effectiveness in treating frequent episodic or chronic tension-type headaches, indicating its potential value as a treatment approach (Linde et al., 2016). Furthermore, studies have explored cervical headaches, cluster headaches, neuralgia headaches, occipital neuralgia, secondary headaches due to stroke, secondary headaches due to hypertension, and other diseases. While there have been published papers, further exploration of these conditions and systematic research is still required.

#### 4.2.2 Diverse selection of acupuncture methods, with acupuncture needle as the mainstay

Acupuncture and moxibustion therapy have a variety of stimulation methods, providing a variety of options for headache treatment, of which acupuncture is the majority. Filiform needle acupuncture and electroacupuncture are the most common needling methods for headache treatment. Filiform needle acupuncture is widely applied, benefiting from extensive scientific research support, accumulated clinical evidence, personalized treatment advantages, and a relatively low risk of side effects. It plays a crucial and significant role in clinical practice. Next is electroacupuncture based on filiform needle acupuncture. Compared to filiform needle acupuncture alone, electroacupuncture offers advantages such as repeatable operations, controllable stimulation intensity, and excellent analgesic effects. It is increasingly recognized and applied by researchers and medical professionals. Furthermore, literature reports also address therapies like moxibustion, warm needle acupuncture, scalp acupuncture, eye acupuncture, ear acupuncture, laser acupuncture, acupoint injection, small needle knife therapy, and acupuncture combined with tuina massage for headache treatment. However, these studies lack substantial sample sizes and high-quality research evidence to establish their therapeutic advantages. Therefore, future research employing large samples, multicenter, and high-quality clinical research designs will help clarify the therapeutic advantages of these methods, identify their most suitable symptomatic characteristics, and further promote the development and application of acupuncture therapy in headache treatment.

#### 4.2.3 Acupuncture: safe, effective, and promising in acute pain management

The safety and effectiveness of acupuncture for headaches have always been a focus. Acupuncture treatment for headaches is associated with high participant satisfaction and a low likelihood of adverse events, making it considered a very safe and effective intervention (MacPherson, 2004). For patients unwilling or unable to use medication for prevention, particularly children or adolescents, acupuncture for headache treatment can be recommended (Doll et al., 2019). Moreover, a research suggested acupuncture as a potential non-pharmacological approach for managing acute pain in pediatric emergency departments (Tsai et al., 2018). Another research found that acupuncture is an effective strategy for treating acute pain and may help prevent or reduce opioid dependency (Nielsen et al., 2022a). The effectiveness of acupuncture in the management of acute pain has been validated, establishing it as a crucial component of comprehensive acute pain care. Therefore, it is necessary for future research to conduct pragmatic, multicenter, well-defined acupuncture randomized controlled trials in emergency departments to clarify the dosage and universality of acupuncture treatment for acute pain (Nielsen et al., 2022b).

#### 4.2.4 Acupuncture’s bidirectional and holistic regulation of pain and emotion

With the continuous expansion of the research field of acupuncture analgesia, researchers have moved beyond merely focusing on exploring the role of acupuncture in regulating pain sensation. Currently, the interaction between acupuncture, pain, and emotional disorders has become a hot topic of research. Numerous studies have revealed that chronic pain and psychological health disorders share common neurobiological mechanisms (Nalini Vadivelu et al., 2017); the application of functional imaging technology further demonstrates that brain regions associated with pain-related emotions and perceptions overlap with those affected in patients with depression (Ehnvall et al., 2009). In terms of treatment, psychological interventions and antidepressants are generally beneficial for pain management (Hooten, 2016). These evidences support a close interrelationship between chronic pain and emotional disorders. Acupuncture can exert a bidirectional and holistic regulatory effect on both aspects. Not only can it alleviate pain, but it can also regulate emotional states. A retrospective cohort study found that acupuncture can not only reduce the severity of headaches in migraine patients but also lower the risk of depression and anxiety (Liao et al., 2020). For chronic pain, acupuncture has been shown to improve pain and psychological disorders (Lin et al., 2020). Additionally, the role of acupuncture in regulating emotions to facilitate pain relief cannot be overlooked. Studies indicate that a good mental state can reduce the number of days migraine patients suffer from headaches, suggesting that the patient’s emotional state is one of the most important non-specific factors in improving the analgesic effect of acupuncture (Pokladnikova et al., 2019). Further research reveals that acupuncture can regulate the brain’s reward/motivation circuits to modulate pain responses (Lee et al., 2015). A study shows that acupuncture can modulate the activity of the brain’s limbic lobes and subcortical structures. The limbic system is a set of key structures in the brain responsible for processing emotions and memory, including areas such as the prefrontal cortex, anterior cingulate cortex, amygdala, hippocampus, and hypothalamus (Hui et al., 2000). When acupuncture achieves “deqi,” these areas exhibit negative activation, and this phenomenon is closely related to the brain’s endogenous pain regulation system (Hui et al., 2005). Another study further elucidated that acupuncture can alleviate pain aversion by promoting the release of opioid substances and 5-HT in the brain’s reward/motivation circuit and by upregulating the expression of GluA1, mGluR1, and GABAB2 proteins in the amygdala, thereby exerting an analgesic effect at the holistic level (Hu, 2019). This is akin to the concepts in traditional Chinese medicine of “alleviating pain and tranquilize” and “regulating the spirit to relieve pain.” Therefore, the mechanisms by which acupuncture treats headaches and emotional disorders warrant further in-depth exploration in the future.

#### 4.2.5 Modern mechanistic research on acupuncture treatment for headaches: brain functional connectivity and neuroimaging techniques

The modern mechanism of acupuncture treatment of headache has been more studied in recent years. Scholars have observed the connection between headaches and the brain, and with the aid of neuroimaging techniques, investigating brain functional connectivity has emerged as a new research focus. Functional connectivity refers to the synchronized neural activity between different brain regions under specific tasks or states, which can be detected and studied using various neuroimaging techniques such as functional magnetic resonance imaging (fMRI) and electroencephalograph (Zhao et al., 2023). More and more studies suggest that functional connectivity networks are closely linked to acupuncture mechanisms, with central integration playing a pivotal role in acupuncture mechanisms (Cai et al., 2018). Supported by fMRI technology, studies discovered that abnormal functional connectivity of the thalamus may be a mechanism underlying the onset and development of migraines (Tian et al., 2021). Another study demonstrated that migraines’ typical pathological feature is abnormal pain processing, with significant functional connectivity abnormalities in the frontal, parietal, and limbic regions (Cao et al., 2022). Therefore, acupuncture can alleviate headache symptoms by restoring pain processing function and regulating pain sensation. Current research proposed that acupuncture might simultaneously regulate the resting state-functional connectivity of two pain-modulating regions—the amygdala and the middle cingulate cortex (Liu et al., 2022). The middle and superior temporal gyrus could be key nodes associated with multisensory processing relevant to acupuncture treatment for migraine regulation. Compared to the period before 2020, the field predominantly relied on clinical trials to demonstrate the effectiveness of acupuncture for treating headaches without clearly understanding its underlying mechanisms. Through the use of neuroimaging techniques, it is now possible to precisely identify the specific brain area affected by acupuncture treatment for headaches. Such research exploring the relationship between acupuncture and functional connectivity is of significant importance for gaining deeper insights into disease mechanisms and developing more effective treatment methods, which in turn aid in early diagnosis, treatment, and disease assessment.

Moreover, imaging technology features assist in predicting the effects of acupuncture. Yang XJ suggested that pre-treatment brain structure could be a new predictive indicator for acupuncture treatment outcomes in migraine without aura (Yang et al., 2020). One study proposed that baseline low-frequency fluctuations in brain regions associated with cognitive pain regulation could predict future improvements in headache intensity in acupuncture-treated patients (Zhengjie et al., 2020). Another finding presented the potential and effectiveness of utilizing machine learning techniques and individual patterns of spontaneous brain activity to forecast the outcomes of acupuncture treatment for migraine without aura (Yin et al., 2020). Hence, with the continuous development of neuroimaging technology, the prospects for studying the relationship between headaches and brain functional connectivity will become even more promising.

## 5 Strengths and limitations

This study investigated the developmental trends of acupuncture treatment on headaches through bibliometric analysis methods. Visualized network diagrams presented various collaborative networks, providing an intuitive depiction. The study analyzed the current state, research hotspots, strengths, and prospects from multiple aspects, including headache types, acupuncture stimulation methods, safety, efficacy, and mechanisms of action. By reading this paper, researchers can quickly identify representative and high-quality research literature, recognize scholars and institutions contributing to the field, and enhance their understanding of the current development status and prospects in this area, promoting collaboration and communication. However, there are certain limitations to this study. Our study solely encompasses English literature from the WoS Core Collection Database, excluding literature from Chinese or other English databases. This omission may impact the study’s conclusions. Furthermore, this study primarily emphasizes quantitative analysis, allocating less attention to qualitative analysis. Consequently, a comprehensive discussion of the specific therapeutic effects and mechanisms associated with various types of acupuncture therapy for related diseases is beyond the scope of this research.

## 6 Conclusion

In conclusion, acupuncture treatment for headaches has demonstrated effectiveness and is characterized by a stable and positive development trend in this field. Research primarily centers around acupuncture’s role in preventing and treating various types of headaches, adapting different acupuncture techniques to specific conditions, and assessing acupuncture’s safety and efficacy. The study of acupuncture’s mechanisms of action primarily focuses on elucidating its bidirectional and holistic regulation of pain and depression and its modulation of brain functional connectivity using neuroimaging techniques. Future research in this field should encompass the advantages of acupuncture in treating different types of headaches, the specific types of headaches targeted by different acupuncture methods, and the modern mechanisms of acupuncture treatment for headaches.

## Data availability statement

The original contributions presented in the study are included in the article/supplementary material, further inquiries can be directed to the corresponding author.

## Author contributions

SZ: Conceptualization, Writing – original draft, Writing – review & editing. SH: Conceptualization, Writing – review & editing. YL: Data curation, Writing – review & editing. WL: Data curation, Writing – review & editing. FZ: Data curation, Writing – review & editing. CW: Data curation, Writing – review & editing. FM: Data curation, Writing – review & editing. XH: Conceptualization, Formal Analysis, Writing – original draft, Writing – review & editing.
